# Advancing Coalition Health Equity Capacity Using a Three-Dimensional Framework

**DOI:** 10.1089/heq.2018.0063

**Published:** 2019-04-27

**Authors:** Paula Tran Inzeo, Brian D. Christens, Amy Hilgendorf, Allison Sambo

**Affiliations:** ^1^ Population Health Institute, University of Wisconsin-Madison, Madison, Wisconsin.; ^2^ Department of Human and Organizational Development, Vanderbilt University, Nashville, Tennessee.; ^3^ Department of Civil Society and Community Studies, University of Wisconsin-Madison, Madison, Wisconsin.

**Keywords:** capacity building, health planning, health promotion, public health practice, health equity practice, socioeconomic factors

## Abstract

**Purpose:** We examined coalition health equity capacity using a three-dimensional conceptual framework in a 3-year study (2011–2014) of 28 local coalitions engaged in health promotion.

**Methods:** Coalition health equity capacity was defined according to (1) conceptual foundations, (2) collective action and impact, and (3) civic orientation. This framework was used to qualitatively assess progress in capacity building efforts at two time points. Coalition materials and archival documents were analyzed qualitatively for indications of each dimension of coalition health equity capacity.

**Results:** The overall cohort of coalitions was initially determined to be near mid-range in conceptual foundations, above mid-range on collective impact, and below mid-range on civic orientation. As part of ongoing training and technical assistance, coalitions were offered examples of high coalition health equity capacity in each dimension. At time point two, growth in health equity capacity was observed in a majority of coalitions.

**Conclusions:** These findings indicate that a multidimensional approach to coalition health equity capacity can be useful for both analysis and practical purposes of community capacity building, which may, in turn, produce long-term gains in health equity.

## Introduction

Advancing health equity is an international priority.^1^ The World Health Organization considers health a human right and defines it as “a state of complete physical, mental, and social well-being and not merely the absence of disease or infirmity.”^2^ Similarly, Healthy People 2020 identifies achieving health equity, eliminating disparities, and improving the health of all groups as one of four overarching goals.^3^

It is widely agreed upon that to work toward health equity, social determinants of health (SDoH) need to be addressed; however, it is less clear what capacities are needed among practitioners to advance health equity. Efforts in the United States have provided resources for communities to include equity in chronic disease prevention. This includes the Centers for Disease Control and Prevention (CDC) Community Transformation Grants,^4^ which aimed to maximize health impact through prevention, advance health equity, and use and expand the evidence base.

Wisconsin, a state with stark racial health disparities,^5^ was one of 68 CDC Community Transformation Grantees and established the initiative, Transform Wisconsin (TWI). In year 1 of TWI, 30 grants were awarded to 28 local coalitions to implement evidence-based policy, systems, and environmental (PSE) changes around tobacco, active living, and food systems. This article describes a study of the health equity specific goals of this project: to build health equity capacity of coalitions and increase/enhance health equity strategies within chronic disease primary prevention efforts. After 2 years, TWI investments resulted in 145,632 new students having new or expanded healthy farm to school food options, 33,670 new students were more active, and 10,577 new people were living in smoke-free housing.^6^

### Background

Health inequities are differences in population health status that are patterned and systemic. They are avoidable and unfair.^7^ Accordingly, many are calling for action on the SDoH to reduce health inequities.^8–10^ In contrast, health equity requires opportunities for all people to achieve their full health and human potential regardless of social identity or socially prescribed disadvantages.^11^

Coalitions can be mechanisms for addressing SDoH in pursuit of health equity.^12,13^ A coalition is a formal association of organizations and individuals that collaborate on a common goal.^14^ Coalitions have successfully addressed health outcomes resulting from social, political, and economic precursors.^12,15^ Coalitions focused on creating change through building community capacity are particularly well positioned to address equity.^16^

Health equity efforts, like other public health issues, are often siloed. Separate offices, programs, and staff specifically dedicated to equity or related topics such as minority health are evidence of this. Issues of equity, however, cut across and affect many public health concerns. Targeted efforts are important, yet it is critical that efforts are connected across issues and practitioners have the capacities to address equity across contexts.^17^

To reduce health inequities, it is necessary to target the SDoH as well as inequitable processes that maintain them through community capacity building, community organizing, and civic engagement.^18^ These approaches are key to addressing systematic exclusion of certain communities from decision-making processes, which contributes to health inequities. Despite agreement on the need to address SDoH, competencies to guide the development of practitioners, organizations, and coalitions to comprehensively address root causes of health do not presently exist.^19^ This study reports on our development and testing of conceptual and empirical tools for monitoring coalition health equity capacity.

### Coalition health equity capacity

To build and assess coalition health equity capacity, we drew on public health literacy and critical health literacy concepts. Public health literacy is the ability to understand and act upon information needed to make beneficial public health decisions.^20^ Critical health literacy is similar and includes skills for taking action at both individual and community levels.^21,22^ Given the key role coalitions have played in health promotion, we built on this literature to define coalition health equity capacity as the degree to which organizations understand, have the skills, orient themselves toward, and implement strategies to advance equity. Accordingly, three dimensions of health equity capacity were identified: (1) conceptual foundations, (2) collective action and impact, and (3) civic orientation. Coalitions with high levels of health equity capacity, we hypothesized, would have and utilize the understandings and skills of each of these inter-related dimensions in their work.

First, conceptual foundations refer to an understanding of the drivers of health and equity, historical contexts in which equity is embedded, and roles power has played in shaping outcomes. Groups with high capacity on this dimension should evidence understanding of social constructs that have and continue to systematically devalue individuals and communities, particularly communities of color and low-income individuals.^19^ Competencies can be observed by a command of health equity principles, formal inclusion of equity language into strategies, through implementation of strategies, reflection, and demonstrated translation of equity language in work plans and actions. Assumptions include socio-ecological perspectives, health as a collective public good, that equity benefits everyone, and that tackling health inequities requires remedying social injustices in systems and structures.

Second, collective action and impact describes coalitions' ability to mobilize diverse multi-sector and multi-level alliances to act in alignment for health improvement. Stakeholders include residents, grassroots community organizations, and professional and governmental agencies. Collective impact is a model that outlines observable conditions for coalition success, including the presence of a backbone agency or infrastructure, commitment to a common agenda, continuous communication, use of shared measures, and mutually reinforcing activities.^23^ Coalitions with these characteristics create spaces for multidisciplinary learning to occur while seeking to address the complexity of determinants of health.^24,25^

Third, civic orientation describes a group's ability to address root causes of health inequities through actions in civil society. Groups with high capacity on this dimension authentically engage and work with communities most impacted by inequities to build power to affect change.^26^ This is often observed in grassroots organizations when new members and leaders are continuously recruited to the organization. In such groups, skill building and leadership development are ongoing priorities; the group builds broad, strategic relationships, and partners are engaged in changing conditions of power and decision making.^27,28^ This competency indicates that health equity practitioners need to be agents in developing capacity and organizing resources to ensure all people can and do participate in social and political processes.^19^ Civic orientation directly and indirectly drives health equity as benefits have been observed related to community conditions and to behavioral, psychosocial, civic, and health outcomes among those who participate.^29,30^

The three dimensions above capture the knowledge, skills, processes, and actions needed for groups to build capacity and advance health equity. When these components are understood and enacted, deep community engagement is occurring, the work is actively confronting power, a socio-ecological perspective is employed, and the work of organizations is oriented toward root causes and structural change to promote health equity. This study applied this three-dimensional conceptual model of health equity capacity as a framework guiding technical assistance (TA) and assessment of coalition capacity.

## Methods

The three-dimensional conceptual framework was used to assess health equity capacity among TWI coalitions. Because little empirical evidence exists on including a health equity lens in PSE change work, this research was exploratory and designed to inform public health initiatives that aim to address health inequities. Our team's work straddled research and practice. As data were gathered, feedback loops to community practitioners were used to ensure that quality improvement processes occurred with all partners.

### Participating coalitions

TWI was a 3-year (2011–2014) statewide effort to implement PSE change related to chronic disease prevention. All 28 funded TWI coalitions participated in this project. Their work focused on implementing evidence-based PSE changes, defined by reputable sources such as the CDC and the Community Preventive Services Task Force, to support active living, increasing access to healthy food, and smoke-free environments. Coalitions represented 25 counties geographically distributed across Wisconsin (a state comprised of 72 counties). Eight of the 10 most populous counties in Wisconsin were represented and 12 counties met the CDC's “rural” classification. Local health departments were fiscal agents for about half of the coalitions; other fiscal agents included other government agencies, nonprofits, and cooperative extension offices. All coalitions included cross-sector agency partners at varying stages of development—from emergent to mature.

### Approach and data collection

All coalitions were provided opportunities to participate in six health equity trainings. The first three trainings corresponded with the dimensions of health equity capacity. These were mandatory and took place during year 1 of the coalitions' grants. The last three addressed common issues coalitions identified when incorporating equity into their work and took place during year 2 of the coalitions' grants. After year 1, coalitions were invited to participate in tailored TA. Twelve of the 28 coalitions took advantage of the opt-in TA. Opt-in TA included an assessment, an initial meeting to identify TA needs and goals, drafting a scope of work, and then the delivery of the work identified.

Given that health equity capacity of coalitions is an exploratory area of study, the evaluation utilized a process of qualitative inquiry and analysis^31,32^ to examine and compare the health equity efforts of these coalitions. Throughout the course of the project, coalitions submitted a variety of archival documents about their coalitions' work each quarter, including governance documents, websites, strategic plans, grant work plans, meeting agendas, and meeting minutes. Additionally, coalition leaders submitted responses to open-ended questions about their health equity efforts and experiences as part of the project's regular monitoring activities. Along with documentation of interactions with the TA team, these materials were included as sources of qualitative data about the health equity efforts of TWI coalitions over the course of the project.

### Analysis

Data were uploaded into NVivo v.10, organized by coalition and by project quarter and year, reviewed, and cleaned for any transpositions or other errors. Three team members thematically analyzed the data,^33,34^ first deductively anchored in the three dimensions of health equity described above. Team members then engaged in inductive or emergent coding to facilitate discovery and further analysis of codes that had not been anticipated by earlier deductive codes.^33^ This process added codes around “missed opportunities” and “coalition member participation.” During this phase, team members coded the same set of coalitions (one-third of the full sample) to compare analyses, discuss discrepancies, and thereby refine and finalize the coding scheme (see Table 1 for a summary of the final coding scheme). The remaining analytic process was coordinated so that the materials for each coalition were reviewed and analyzed by at least two team members and any remaining discrepancies in coding were resolved through discussion and documented in memos.

**Table 1.** Coding Scheme

Code	Sub-code	Description
Conceptual foundations	Social determinants of health	Reflects understanding of social determinants of health
	Systems	Reflects understanding and attention to systems or socio-ecological factors
	Language	Formal inclusion of health equity language in coalition materials
Collective impact	Common agenda	Participants reflect a shared vision for change, including a common understanding of the problem, a joint approach, and agreed-upon actions
	Shared measurement	Collecting data and measuring results consistently across participants to ensure efforts remain aligned and participants hold each other accountable
	Mutually reinforcing activities	Participant activities are differentiated while still coordinated through a mutually reinforcing plan of action
	Continuous communication	Consistent and open communication to build trust, assure mutual objectives, and create common motivation
	Structural support	Creating and managing collective impact through a separate organization or dedicated staff to serve as the backbone for the coalition and coordinate participating organizations
Civic orientation	Engagement	Authentic engagement with populations experiencing health inequities
	Empowerment	Efforts to empower populations experiencing health inequities, including skill-building activities and leadership development
Missed opportunities		Indicates events/interactions/situations in which health equity work could have been better integrated
Coalition member participation		Demonstrated active engagement of coalition members

After this coding process, the team began comparing coalitions. Returning to the three dimensions of health equity, the full team reviewed results for each coalition and discussed how results indicated the coalition's health equity capacity in each dimension per year of the grant, from “No evidence of health equity work” on one end and “Addressing health equity in an ideal way” on the other end of the spectrum. Based on these dimensional placements, coalitions were then grouped into one of four groups with other coalitions whose capacities were ranked similarly. These results were summarized visually and narratively into individual reports provided to each coalition, including recommendations for continued health equity capacity development.

## Results

**FIG. 1.**
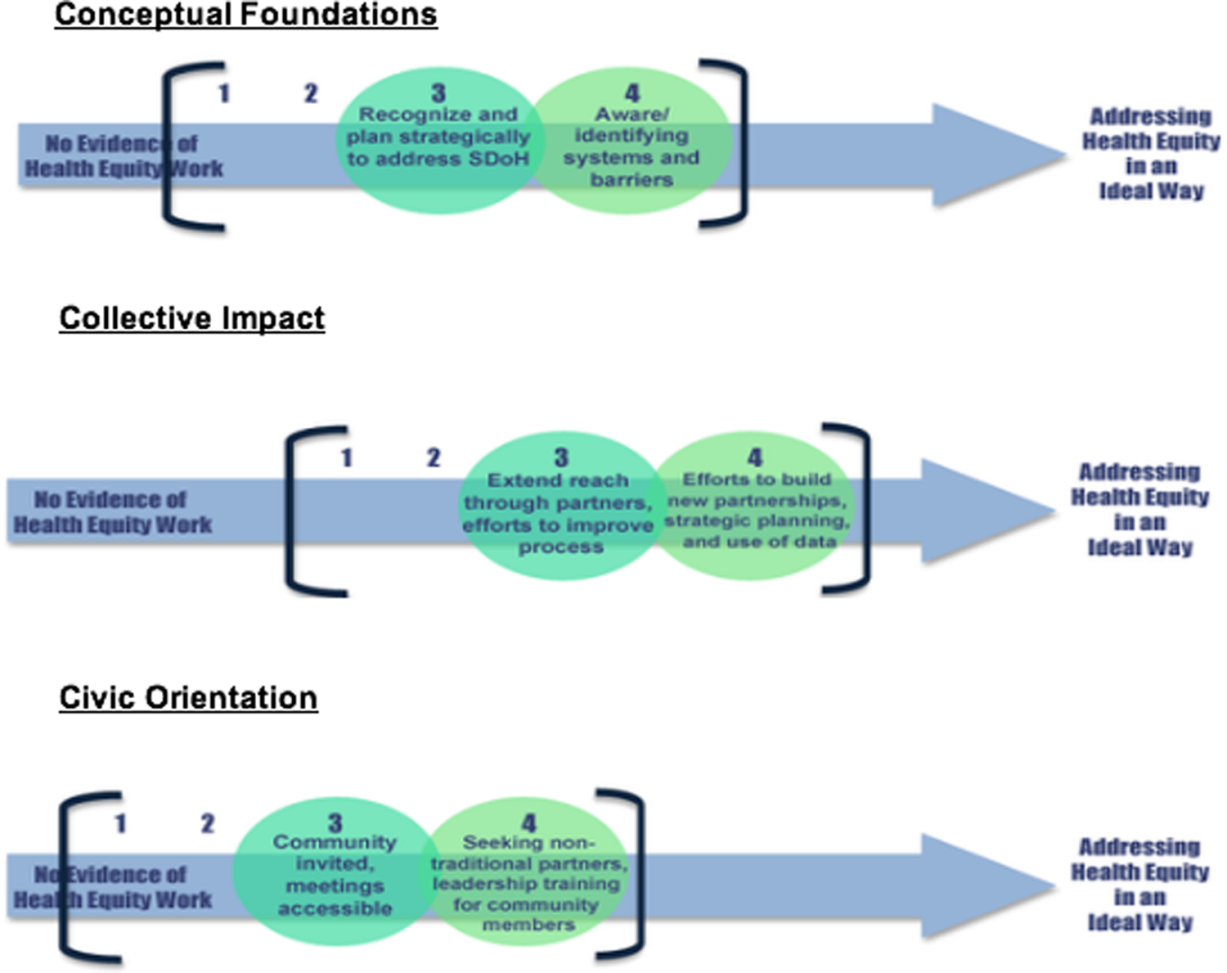
Dimensions of health equity capacity spectrums, shows the three spectrums created to depict coalition health equity capacity rankings relative to the cohort of coalitions.

**FIG. 2.**
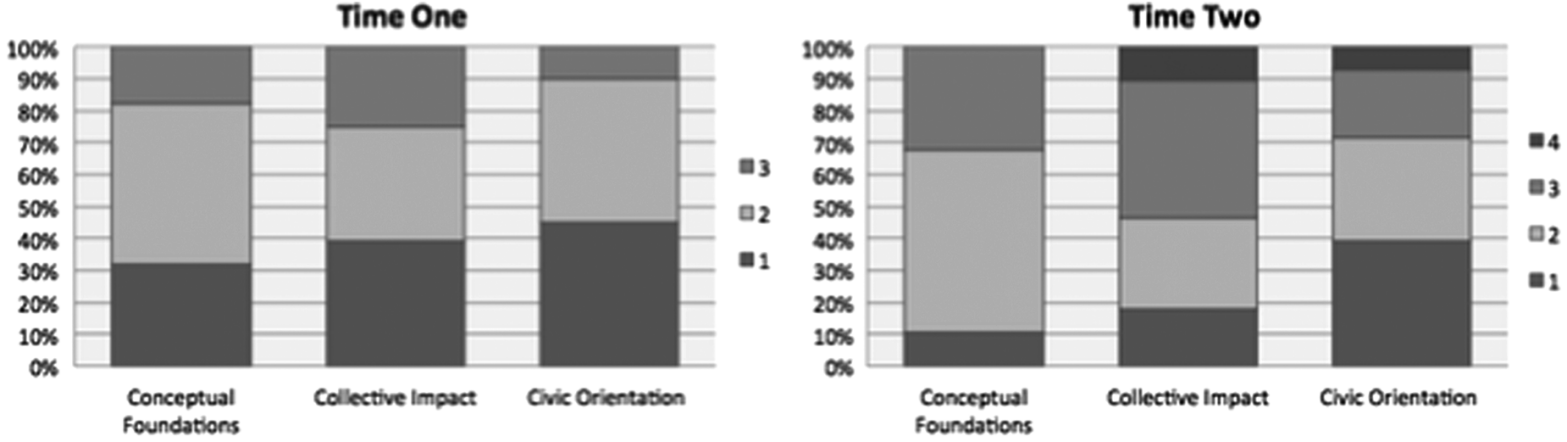
Changes in health equity capacity of 28 local Wisconsin coalitions, shows two bar graphs, the one on the left at time one and the one on the right at time two that shows the changes in coalition health equity capacity rankings.

Visual spectrums approximated and represented the assessment of each dimension of health equity capacity (Fig. 1). Each spectrum included “No evidence of health equity work” on the far left to “Addressing Health Equity in an Ideal Way” on the far right. Based on coding, the median of the initial cohort of coalitions was determined to be near mid-range (i.e., some evidence of health equity work) on conceptual foundations, above mid-range on collective action and impact, and below mid-range on civic orientation. To offer coalitions a sense of how their results compared to others, they were placed in groupings^1–4^ with other coalitions whose capacities were similar. They were also offered descriptions of the best work observed from the cohort of coalitions on each dimension. No coalition achieved a four in the first round. Coalitions made progress beyond what was initially observed, and some achieved a rating of four during the second round of assessment. Growth in health equity capacity was observed in 20 of the 28 coalitions. Five in at least one dimension, 13 in two dimensions, and two grew across all three dimensions (Fig. 2).

### Conceptual foundations

Analyses indicated that most groups understood what the SDoH were and their impacts on inequities. There was less understanding of the role of power imbalances between social groups and within institutions in allocating SDoH. During round one, we observed a number of coalitions identifying populations experiencing negative outcomes associated with inequities in their communities, often through the use of data. These populations included specific neighborhoods, racial/ethnic groups, elderly and aging residents, low-income individuals and families, individuals with disabilities, and/or youth. Coalition engagement with these priority communities was a common theme within these results. About one quarter of the coalitions were working to target strategies toward these priority communities.

By round two, almost all coalitions had identified priority communities, and most were targeting some strategies toward these populations. Deeper understandings of the SDoH were observed through the number and quality of formal coalition conversations on topics related to health equity (e.g., living wages, discrimination, and community engagement). Observations of multi-pronged strategies and systems approaches along with strategic partnerships illustrated gains in capacity for implementing health promotion and disease prevention strategies informed by a socio-ecological framework. A small number of coalitions also began to use tools such as participatory photomapping, health impact assessment, and data related to social determinants for increasing understanding and action around inequity.

### Collective action and impact

During round one, it was observed that all coalitions had some structure to support collective work. Small numbers were working with multi-sector partners and three coalitions incorporated equity into their goals.

During round two, almost all coalitions increased the sophistication of their collaborative infrastructure. This was observed through increased strategic planning, increased use of data in strategic development and evaluation, increased use of specific tools such as asset mapping to understand local coalition context and partners, increased efforts to build coalition capacity through trainings and workshops, and increased internal and external communications. Coalitions' partnerships increased in depth and diversity as groups began to engage and work with historically nontraditional partners for local public health.

### Civic orientation

Overall, coalitions demonstrated a relatively low level of civic orientation. By the end of the project, many were just beginning to understand and find ways to explore deepened partnerships with priority communities. During round one, the extent of civic orientation was demonstrated by coalitions targeting educational and outreach activities toward priority communities in their geographic areas. Coalitions were also working to build partnerships with agencies that represented or served affected communities. For example, two coalitions were working to build community leadership capacity and one coalition was trying to build deeper relationships with community members.

During round two, coalitions evidenced increased specificity around discussions of health equity and worked to tailor the ways they worked with priority communities. Tailored activities involved identifying shared interests between coalitions and these communities, providing training and capacity building, brokering access to needed resources, enhancing strategic partnerships, and creating new roles within coalition structures.

### Health equity capacity

Across all dimensions, the cohort of coalitions was observed to have strong understanding of SDoH. In the area of collective action and impact, they exhibited increasingly well-established and effective structures to support collective action. Coalitions demonstrated the least understanding and work in areas that require a critical analysis of power and a sense of strategies to address imbalances in power at multiple levels, creating a gap in ability to target structural root causes of inequity. This indicates a need to invest in efforts to build capacity in related areas that mainly fall in the dimension of health equity capacity that we term civic orientation—a relatively new concept within public health practice.

## Discussion

This study applied a novel framework for advancing coalition health equity capacity in scholarship and practice. The study team served as TA providers to build coalition capacity and as an action-oriented evaluation team. As data were collected and analyzed, findings were shared with coalition leaders to inform ongoing development and “member check” the data. The multiple roles of the study team shaped the way information was shared with the coalitions, ensuring greater accessibility and understanding, as well as extending an open invitation for further discussion and feedback. This process of structured qualitative analysis paired with tailored TA and training likely played some role in increasing coalitions' health equity capacity. This framework for integrated TA and assessment may be useful to other efforts to orient coalitions' work toward equity.^12–16,35^

There are a number of limitations to this study. While analyses make clear that coalitions deepened their capacity to engage in equity-oriented work during the course of the study, it is difficult to precisely assess the amount of growth since data analyses were primarily descriptive. Quantity and quality of reported data also varied across coalitions, making it difficult to have a uniform picture of each coalition. Future research may build on this work to identify new tools for assessment of coalition health equity capacity.

It is also important to note that inequities continue to produce large disparities in health in Wisconsin, and continued efforts to address SDoH will be required at multiple levels (i.e., changes in local, state-level, and federal policies and systems). Building local coalitions' capacity to pursue such changes is one important avenue for action among many that are needed.^1,8,10,19^ Studies with longer time horizons are needed to assess the impacts of multiple changes in SDoH on population health outcomes.

## Conclusion

The analyses presented in this study advance the operationalization of incorporation of health equity into coalition-based health promotion efforts. In this sense, it provides a response to a struggle heard broadly from public health practitioners.^10,19^ We were able to descriptively assess three dimensions of health equity, which can be used to inform future coalition capacity building and measurement of coalition health equity capacity. Given the observed changes in practice over the project period, building and assessing health equity capacity of coalitions using the three-dimensional framework presented here is a promising strategy to shift coalition efforts toward progress on health equity.

## Abbreviations Used

CDC: Centers for Disease Control and Prevention
PSE: policy, systems, and environmental
SDoH: social determinants of health
TA: technical assistance
TWI: Transform Wisconsin
